# Supplementary material for: Content-rich biological network constructed by mining PubMed abstracts
Source: BMC Bioinformatics. 2004 Oct 8;5:147. doi: 10.1186/1471-2105-5-147 (PMC528731; doi:10.1186/1471-2105-5-147)
Supplement: Additional File 5 — The original Chilibot query results of the term "long-term potentiation (LTP)" and 22 other terms, limiting the latest references analyzed to the years 1990, 1995, 2000, and 2004. [file 1471-2105-5-147-S5.bz2 › chilibotAdditionalFile5/ltp1995/html/NMDA_TAU.html]

 


 **NMDA** and **TAU** 
  
Found 42 abstracts in PubMed,  **30 abstracts were retrieved and analyzed**.  


---

 Search Google  |
 PDF files only 
|  EDU domain only 

---

**Interactive relationship** (e.g. stimulation, inhibition, etc)

- The KCl  **NMDA**  induced dephosphorylation of  **tau**  was blocked by the non competitive  **NMDA**  receptor antagonist MK801.  Ref: 7619080 Biochem J, 1995
- Addition of KCl  **NMDA**  to the slices resulted in a 40% decrease in 32P incorporation into  **tau** .  Ref: 7619080 Biochem J, 1995
- The principal effect of BIC Mel 10 100 microM on  **NMDA**  channels was a dose dependent decrease in mean channel open time  **tau**  o, accompanied by the introduction of a new closed time  **tau**  B of 14.0 3.5 msec mean standard deviation.  Ref: 1534141 Mol Pharmacol, 1992
- Preliminary single channel experiments performed under conditions conducive to trapping of an open channel blocker at its binding site indicated that the effect of BIC Mel on  **tau**  o persisted after the removal of the blocker, consistent with use dependence of the dissociation of BIC Mel from the  **NMDA**  receptor channel complex.  Ref: 1534141 Mol Pharmacol, 1992
- Pretreatment of the rat brain slices with Cyclosporin A completely abolished the dephosphorylation of  **tau**  induced by the addition of KCl  **NMDA** .  Ref: 7619080 Biochem J, 1995
- Modulation of  **tau**  neuronal expression induced by  **NMDA** , non  **NMDA**  and metabotropic glutamate receptor agonists.  Ref: 7541298 Neurodegeneration, 1995
- Acute and chronic  **NMDA**  treatment induced a concentration dependent increase in intraneuronal  **tau**  immunoreactivity.  Ref: 7541298 Neurodegeneration, 1995
- Determine the involvement of the calcium calmodulin dependent phosphatase, calcineurin, in the KCl  **NMDA**  induced dephosphorylation of  **tau** , slices were pretreated with the calcineurin inhibitor Cyclosporin A.  Ref: 7619080 Biochem J, 1995
- It is concluded that both 1 the concentration of Asp, Glu, Gly and  **Tau**  and 2 the changes induced by  **NMDA**  receptor activation or blockade are different at 16 vs. 30 days of age.  Ref: 8413833 Neuroendocrinology, 1993
- Increasing intracellular concentrations of calcium by treatment of the brain slices with the glutamate analogue  **NMDA**  in depolarizing conditions 55 mM KCl resulted in dephosphorylation of  **tau** .  Ref: 7619080 Biochem J, 1995
- ... addition of KCl or  **NMDA**  alone had no effect on  **tau**  phosphorylation.  Ref: 7619080 Biochem J, 1995
- We have analysed changes in  **tau**  protein immunoreactivity in rat embryonic neurons degenerating in response to treatment with N methyl D aspartate  **NMDA** , non  **NMDA**  and metabotropic agonists.  Ref: 7541298 Neurodegeneration, 1995
- At 80 mV the  **NMDA**  receptor mediated EPSC latency = 2.7 ms displayed a slow rise time 10 90%, 5.8 ms and exhibited a biexponential decay time constant of fast component of decay  **tau**  s = 27.6 ms, time constant of slow component of decay  **tau**  s = 147.4 ms.  Ref: 7823088 J Neurophysiol, 1994

**Parallel relationship** (e.g. studied together, co-existance, homology, etc.)

- Furthermore, the swallowing response of the neurons was depressed by ionophoretic applications of both the broad spectrum EAA antagonist,  **tau**  D glutamyl glycine, and the selective  **NMDA**  antagonist, DL 2 amino 5 phosphonovalerate, and by pressure applications of the preferential non  **NMDA**  receptors blocker, 6 cyano 7 nitroquinoxaline dione.  Ref: 8096425 Brain Res, 1993
- MK 801 pretreatment abolished  **NMDA**  induced changes and reduced  **Tau**  26% and Gly 30% levels.  Ref: 8413833 Neuroendocrinology, 1993
- Alterations in situ in the phosphorylation state of the microtubule associated protein  **tau**  were examined in response to increasing intracellular levels of calcium through N methyl D aspartate  **NMDA**  receptor activation, or activating cyclic AMP cAMP dependent protein kinase cAMP PK, in rat cerebral cortical slices.  Ref: 7619080 Biochem J, 1995
- The N methyl D aspartate  **NMDA**  receptor antagonist D 2 amino 5 phosphonovaleric acid APV, 10 microM significantly decreased mEPSC decay  **tau**  and frequency only at depolarized membrane potentials.  Ref: 7507519 J Neurophysiol, 1993
- BIC Mel 10 100 microM increased the frequency of  **NMDA**  channel opening in a dose dependent manner, offsetting the decrease in  **tau**  o, such that the total time spent in the open state per minute was unchanged, and thus the total charge min through  **NMDA**  channels was unchanged.  Ref: 1534141 Mol Pharmacol, 1992
- Increased  **tau**  immunolabelling during chronic  **NMDA**  toxicity was dramatically attenuated by tetrodotoxin and also by 6 cyano 7 nitroquinoxaline dione.  Ref: 7541298 Neurodegeneration, 1995
- the time constant  **tau**  of the slow component of the decay of postsynaptic currents in the presence of each compound was greater than the control  **tau** , the IC50 of IEM 1754 for inhibition of  **NMDA**  activated whole cell currents was > 20 times larger than its Kd for the open channel, and a transient increase in  **NMDA**  activated whole cell currents was observed after washout of IEM 1754.  Ref: 7535380 Mol Pharmacol, 1995
- The role of  **tau**  aminobutyric acid GABA A receptors and of the N methyl D aspartate  **NMDA**  subtype of excitatory amino acid receptors was studied in the pentylenetetrazol PTZ kindling model.  Ref: 1323663 J Pharmacol Exp Ther, 1992
- The present experiments were designed to study in female rats during sexual maturation 1 the hypothalamic release of aspartate Asp, glutamate Glu and glycine Gly which are the excitatory amino acids EAAs involved in  **NMDA**  neurotransmission and of taurine  **Tau** , a putative inhibitory amino acid of GnRH secretion.  Ref: 7898628 Neuroendocrinology, 1995
